# Ginseng Metabolites on Cancer Chemoprevention: An Angiogenesis Link?

**DOI:** 10.3390/diseases3030193

**Published:** 2015-09-02

**Authors:** Chong-Zhi Wang, Yi Cai, Samantha Anderson, Chun-Su Yuan

**Affiliations:** 1 Tang Center for Herbal Medicine Research, Pritzker School of Medicine, University of Chicago, 5841 South Maryland Avenue, MC 4028, Chicago, IL 60637, USA; E-Mails: caiyi219@gmail.com (Y.C.); anderson.samantha6@gmail.com (S.A.); cyuan@uchicago.edu (C.-S.Y.); 2 Department of Anesthesia and Critical Care, Pritzker School of Medicine, University of Chicago, 5841 South Maryland Avenue, MC 4028, Chicago, IL 60637, USA; 3 Committee on Clinical Pharmacology and Pharmacogenomics, Pritzker School of Medicine, University of Chicago, 5841 South Maryland Avenue, MC 4028, Chicago, IL 60637, USA

**Keywords:** American ginseng, *Panax quinquefolius*, angiogenesis, cancer management, metabolites, ginsenoside Rg3, compound K, intestinal microbiome

## Abstract

Cancer is a leading cause of death in the United States. Angiogenesis inhibitors have been introduced for the treatment of cancer. Based on the fact that many anticancer agents have been developed from botanical sources, there is a significant untapped resource to be found in natural products. American ginseng is a commonly used herbal medicine in the U.S., which possesses antioxidant properties. After oral ingestion, natural ginseng saponins are biotransformed to their metabolites by the enteric microbiome before being absorbed. The major metabolites, ginsenoside Rg3 and compound K, showed significant potent anticancer activity compared to that of their parent ginsenosides Rb1, Rc, and Rd. In this review, the molecular mechanisms of ginseng metabolites on cancer chemoprevention, especially apoptosis and angiogenic inhibition, are discussed. Ginseng gut microbiome metabolites showed significant anti-angiogenic effects on pulmonary, gastric and ovarian cancers. This review suggests that in addition to the chemopreventive effects of ginseng compounds, as angiogenic inhibitors, ginsenoside metabolites could be used in combination with other cancer chemotherapeutic agents in cancer management.

## 1. Introduction

Cancer is a leading cause of human death in the United States [1]. The clinical management of cancer invariably involves diverse conventional modalities, including surgery, radiation, and chemotherapy [1,2]. Because commonly used chemotherapeutic agents usually affect processes that occur in all rapidly dividing cells, many normal cells throughout the body that are undergoing active growth and cell division can also be damaged. As a result, chemotherapeutic agents induce significant side effects [3]. To reduce the adverse effects caused by chemotherapeutic agents, in recent years, targeted therapies were developed to interact with specific molecules that are part of the pathways [4].

Tumor growth and progression depend on angiogenesis, a process of new blood vessel formation from a preexisting vascular endothelium. The newly formed blood vessels provide nutrients and oxygen to the tumor, increasing its growth. Inhibiting angiogenesis has been proposed as a potential cancer treatment strategy [5]. Therefore, angiogenesis is considered as a powerful target to suppress tumor growth and metastasis [6,7].

Complementary and alternative medicine (CAM), which covers a wide spectrum of ancient to new-age approaches that purport to expand options for preventing and treating diseases, is gaining more attention for cancer management [8,9,10]. The emergence of CAM represents a natural experiment of huge dimensions, as millions of Americans have begun self-medicating with natural products [11,12]. Natural products have been valuable sources of new therapeutic candidate compounds [13,14,15]; an analysis of the number of chemotherapeutic agents and their sources indicates that nearly 80% of approved drugs are derived from natural compounds [16]. With the advent of high throughput screening technologies, natural products are likely to provide many of the lead structures for the construction of novel compounds with enhanced anticancer properties.

Natural products are most often administered orally so that they are exposed to the trillions of microbial organisms that live in the gut. Previous reports revealed that compound biotransformation resulted from reactions carried out by the enteric microbiome [17]. These studies involved monitoring compound metabolism during the *ex vivo* fecal incubation with a given compound [18], showing that orally ingested natural products can be biotransformed to their metabolites by microbiota in the gut. However, information regarding the role of the enteric microbiome in botanical bioactivity is still limited and this situation obstructs the evaluation of natural products with anticancer potential.

Because ginseng is a very commonly used antioxidant natural product in the U.S, in this review, using American ginseng as an example, we have summarized recent research progress on the anticancer activities of ginseng parent compounds and their intestinal microbiome metabolites, focusing on their angiogenesis inhibitory potentials. Crucial information on ginseng’s interactions with the enteric microbiome was obtained and the contribution of intestinal microbiota to ginseng’s anticancer activity is discussed. Molecular mechanisms involved in the ginseng metabolites’ actions, including those targeted on angiogenesis are discussed.

## 2. American Ginseng is a Commonly Used Antioxidant Botanical

Ginseng is the name of a group of botanicals in the genus *Panax* of the Araliaceae family. Three species in the genus are commonly used as herbal remedies in oriental countries, *i.e.*, Asian ginseng (*Panax ginseng*), notoginseng (*Panax notoginseng*), and American ginseng (*Panax quinquefolius*).

Asian ginseng is distributed in Eastern Asia, including northeastern China, Korea, and the far east of Russia, while commercially available Asian ginseng is cultivated in China and Korea [19,20]. Like Asian ginseng, notoginseng is a Chinese herbal medicine that has a long history of use in Asian countries. Notoginseng is distributed in southwestern China, Burma, and Nepal, and this herb is cultivated commercially in southwestern China [21,22]. The root is the most commonly used plant part of Asian ginseng and notoginseng.

American ginseng is an obligate shade perennial plant native to eastern North America. The commonly used part of the plant is the root, which is harvested after several years of cultivation. Although both the U.S. and Canada have cultivated American ginseng, the largest growing area is in Wisconsin, USA [23,24]. In recent years, American ginseng has also been grown in northeastern China on a small scale, however, studies on the chemical profiles and pharmacological effects of Chinese produced American ginseng are limited [25].

**Figure 1:**
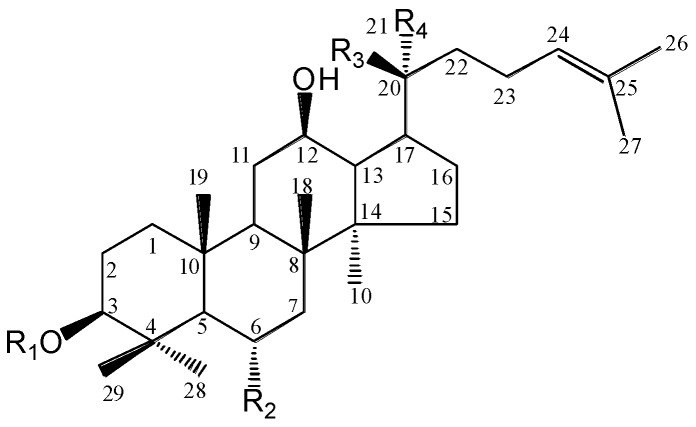
Chemical structures of ginseng saponins in American ginseng. Rg3, F2, and CK are gut microbiome metabolites of PPD group saponins.

It is generally believed that the active compounds in Asian ginseng, notoginseng and American ginseng are triterpene glycosides or dammarane saponins, commonly referred to as ginseng saponins (ginsenosides and notoginsenosides). These ginseng saponins are the major active ingredients in the herb, and their levels can be used to develop quality controls for these herbs [26,27,28]. There are over 50 different known ginseng saponins, and they are characterized by a four trans-ring rigid steroid aglycone skeleton and attached sugar moieties [29]. Based on the aglycone skeleton, ginseng saponins can be divided into the protopanaxadiol group and protopanaxatriol group, except for ginsenoside Ro, which is derived from the oleanolic acid group (Figure 1).

Although all three ginseng plants have both protopanaxadiol group (PPD group) and protopanaxatriol group (PPT group) saponins, their constituent compositions vary. As we know, ginsenosides Rb1, Rb2, and Rd belong to the PPD group, while ginsenosides Re, Rg1, and notoginsenoside R1 belong to the PPT group. The major saponins in Asian ginseng are ginsenosides Rb1, Rb2, and Rg1; those in notoginseng are ginsenosides Rb1, Rd, Rg1 and notoginsenoside R1; and the major saponins in American ginseng are ginsenosides Rb1, Rd, and Re.

American ginseng has a high saponin content, specifically having abundant PPD group saponins [30]. Previous observations suggested that the PPD group ginsenosides, especially those with lesser sugar moieties, showed more potent anticancer activity [31,32]. The higher PPD group saponin content in American ginseng supplied sufficient resources for further chemical or biochemical transformation to produce active anticancer ginsenosides, which will be discussed later.

Several beneficial effects of American ginseng, such as cardioprotection and adjuvant cancer therapy, have been reported [33,34]. These pharmacologic activities are, to a significant extent, considered to be due to the antioxidant properties of this botanical. Our group observed anti-diabetic and anti-obesity activities of American ginseng extract [35,36,37]. These effects are likely related to the anti-oxidant property of the ginseng compounds. In a clinical trial, ginseng extract prevented acute oxidant injury following cardiac reperfusion [38]. Our group has demonstrated that acute oxidant stress induced cardiomyocyte injury was protected by the treatment of American ginseng [39]. For cancer chemotherapy studies, we observed that American ginseng possesses the potential for treating chemotherapeutic agent-induced side effects, which is partly mediated by antioxidant mechanisms [40,41].

**Table:** 

Group	Saponin	R_1_	R_2_	R_3_	R_4_
PPT	Rg_1_	H	*O*-glc	*O*-glc	CH_3_
	Re	H	*O*-glc^2^ → ^1^rha	*O*-glc	CH_3_
	Rh_1_	H	*O*-glc	OH	CH_3_
	20*S*-Rg_2_	H	*O*-glc^2^ → ^1^rha	OH	CH_3_
	20*R*-Rg_2_	H	*O*-glc^2^ → ^1^rha	CH_3_	OH
PPD	Rb_1_	glc^2^ → ^1^glc	H	*O*-glc^6^ → ^1^glc	CH_3_
	Rc	glc^2^ → ^1^glc	H	*O*-glc^6^ → ^1^araf	CH_3_
	Rb_2_	glc^2^ → ^1^glc	H	*O*-glc^6^ → ^1^arap	CH_3_
	Rb_3_	glc^2^ → ^1^glc	H	*O*-glc^6^ → ^1^xyl	CH_3_
	Rd	glc^2^ → ^1^glc	H	*O*-glc	CH_3_
	Rg_3_	glc^2^ → ^1^glc	H	OH	CH_3_
	F_2_	glc	H	*O*-glc	CH_3_
	CK	H	H	*O*-glc	CH_3_

## 3. Biotransformation of American Ginseng Saponins

Bacteria are an important component of the human body. The microbiome is a community of living microorganisms assembled in particular ecological niches of a host that contain trillions of bacterial cells, 10 times more cells than the number of cells constituting the body [42]. A considerable portion (approximately 70%) of this microbial cosmos is localized in the gut, while the colon is the site where the gut microbiota reaches its highest concentration [43].

**Figure 2:**
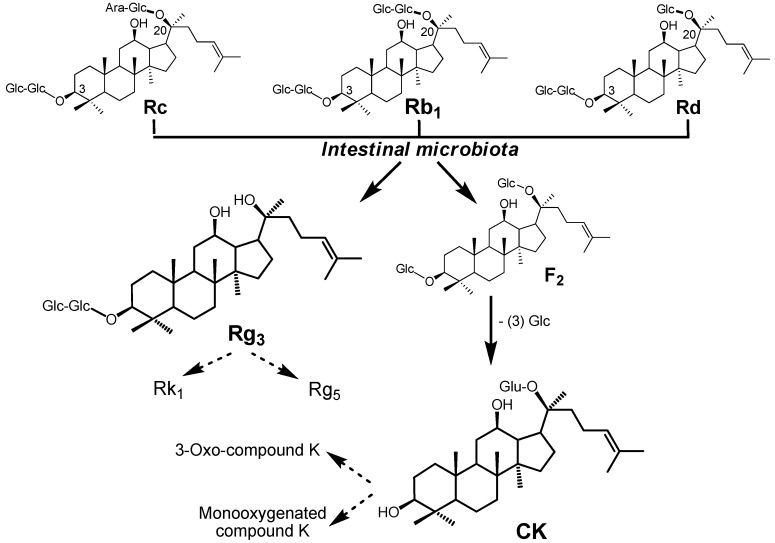
Metabolic pathways of PPD group ginseng saponins converted by the intestinal microbiome.

Like many other herbal medicines, the route of administration of American ginseng is nearly always oral. After oral ingestion, ginseng saponins are metabolized extensively by intestinal microbiota. Although investigations on the comprehensive metabolic profile of American ginseng are very limited because of the chemical complexity and limitation of analytical methods, we recently systemically evaluated the biotransformation and metabolic profiles of American ginseng extract by human intestinal microflora. Using a highly sensitive and selective liquid chromatography/quadrupole time-of-flight mass spectrometry method, 25 metabolites were detected, of which 15 metabolites were derived from original protopanaxadiol saponins, including the three highest ones. This indicated that the PPD-type ginsenosides generated comprehensive biotransformation and were more easily metabolized than other ginsenosides under the same conditions. Several major metabolic pathways can be observed for PPD-type ginseng saponins by human intestinal microbiota such as deglycosylation and dehydration. One way is to selectively eliminate the *C*-3 sugar moieties to produce F_2_ and then compound K (CK) [44,45]. CK can be further converted to 3-oxo-compound K and monooxygenated compound K. Another way is to selectively eliminate the *C*-20 sugar chain to produce ginsenoside Rg_3_ [46]. It is interesting to note that Rg_3_ can be further transformed to Rk_1_ and Rg_5_ by intestinal microflora via dehydration (Figure 2) [46].

The three most abundant metabolites are ginsenoside Rg_3_, ginsenoside F_2_, and CK, which are transformed from ginsenosides Rb1, Rb2, Rb3, Rc, and Rd (Figure 1). The main metabolic pathways of ginseng saponins are deglycosylation reactions by intestinal microflora through the stepwise cleavage of sugar moieties. Subsequent dehydration reactions also occur. Compared to those of the PPT group, PPD group triterpenoids are easy to metabolize.

We recently observed that CK showed significant antiproliferative effects on human colorectal cancer cells, while its parent compound Rb1 did not show any effects at the same concentrations [47]. Thus, ginseng’s bioactivity appears to be highly dependent on metabolism by enteric microbiota. Several microbial species including *Prevotelki oris* appear to have this capability [44,45,48]. However, information on the role of enteric microbiota in ginseng’s cancer chemoprevention is still not clear. Nevertheless, available evidence has suggested that the intestinal microbiota may play an important role in mediating the metabolism and bioactivity of American ginseng.

## 4. Effects of Ginsenoside Metabolites on Cancer Cell Death Induction

Early ginseng anticancer evaluations largely focused on the herb’s parent compounds, *i.e.*, the ginsenosides Rb1, Rd, and Re. The antiproliferative effects of these ginsenosides on different cancer cell lines were evaluated. Unfortunately, in several *in vitro* screenings, even in very high concentrations, these parent compounds did not show obvious antiproliferative activities [31,49].

Orally administered ginsenosides are poorly absorbed, and some appear to require bacterial metabolism to be absorbed and biologically active [50]. After ginseng ingestion, both Rg3 and CK are major metabolites reaching the systemic circulation [29,46,51]. CK possesses very significant anticancer activities compared to its parent compound Rb1 [52]. The IC_50_ of CK for the inhibition of colon cancer cell proliferation was 30–50 µM, suggesting that its antiproliferative effect is greater than that of Rg3 (IC_50_ 100–150 µM), the compound derived from Rb1 via steaming treatment [47,53,54].

Although ginsenoside Rg3 showed moderate antiproliferative effects, Rg3 was approved as a new anti-cancer drug in China [55]. Rg3 has been shown to inhibit NF-κB signaling [56] and enhances the susceptibility of prostate cancer cells to docetaxel and other chemotherapeutics [57]. We observed that the inhibitory effect of Rg3 on colon cancer cells is in part mediated by inhibiting β-catenin/Tcf transcriptional activity and suppressing PNCA expression in colon tumors, thus inducing cancer cell apoptosis [58].

CK induced apoptosis in several tumor cell lines by regulating various signaling pathways, such as the activation of caspase-8 [59] and AMP-activated protein kinase (AMPK) [60,61], suppression of nuclear factor-kappa B (NF-кB) pathways [62] and Janus activated kinase 1 (JAK1)-signal transducer and activator of transcription 3 (STAT3) signaling [63]. A recent study indicated that CK increased generation of reactive oxygen species and activation of c-Jun NH2-terminal kinase signaling pathway, which in turn stimulated autophagy and apoptosis in human colon cancer cells [64]. Our data showed that multiple pathways, including p53/p21, PI3K/Akt, and transforming growth factor beta (TGF-β), were involved in CK induced cancer cell death [65].

## 5. The Role of Ginsenoside Metabolites in Angiogenesis for Cancer Treatment

Anti-angiogenic compounds have emerged as potential agents to be used alone or in combination with other chemotherapeutic drugs for the treatment of cancer. Early studies have revealed that red ginseng extract inhibited tumor metastasis, and active compound ginsenoside Rg3 exhibited a significant decrease in the number of blood vessels oriented toward the tumor mass [66]. As shown in Figure 2, Rg3 is also a major intestinal metabolite from parent PPD group ginsenosides. Rg3 was found to inhibit the proliferation of human umbilical vein endothelial cells.

The vascular endothelial growth factor (VEGF) is an important growth factor that promotes angiogenesis. Overexpression of VEGF results in increased angiogenesis, whereas its suppression results in the inhibition of angiogenesis in both normal and pathological conditions. Increased VEGF expression is associated with solid tumor growth, and its inhibition has been shown to be effective in the control of several cancers [67]. Rg3 suppressed the capillary tube formation of the cells in the presence or absence of VEGF. The VEGF-induced cell chemoinvasions were significantly attenuated by Rg3 [68].

Rg3 also showed anti-angiogenic effects on pulmonary, gastric, and ovarian cancers [69,70,71]. As a potential angiogenic inhibitor, Rg3 processed synergistic effects when it was used in combination with other cancer chemotherapeutic agents, such as gemcitabine, cyclophosphamide, and capecitabine [72,73,74]. The combination use of chemotherapeutic agent plus angiogenesis inhibitor could be a promising strategy in cancer management.

As a major ginsenoside metabolite in the gut, CK showed significant anticancer activity. In early studies, in addition to inducing cancer cell apoptosis, CK also showed antiplatelet and anti-angiogenic activities and caused the suppression of primary tumor growth in a spontaneous metastasis model [45]. MMPs (matrix metalloproteinases), especially MMP-9, involved in extracellular matrix degradation, are crucial for the endothelial cell migration, organization, and angiogenesis [75]. It has been reported that MMP-9 expression levels are significantly higher in tumors than in normal tissue. The upregulation of MMP-9 is implicated in the process of invasion and metastasis of malignancies [76]. CK significantly inhibited the secretion and protein expression of MMP-9. The inhibitory effect of compound K on MMP-9 expression was correlated with decreased MMP-9 mRNA levels and the suppression of MMP-9 promoter activity [77].

Using human umbilical vein endothelial cells (HUVECs), the molecular mechanisms of the anti-angiogenic effects of CK were further evaluated. CK significantly inhibited the migration and tube formation of HUVECs at non-cytotoxic concentrations, reduced the secreted level of VEGF and increased the secreted level of pigment epithelium-derived factor (PEDF) in HUVECs. The anti-angiogenic activity of CK was found to be a result of the inhibition of p38 MAPK and AKT in the HUVECs [78]. Anti-angiogenic effects of CK were also validated by a recently published study [79]. Therefore, the anti-angiogenic effects of CK might have therapeutic potential for controlling the growth and invasiveness of different cancers.

## 6. Conclusions

Anti-angiogenic compounds have emerged as potential agents to be evaluated for the treatment of cancer. Many anticancer agents have been developed from botanical sources. American ginseng is a commonly used antioxidant herbal medicine in the United States. Ginsenoside Rg3 and CK are major ginseng metabolites biotransformed by the enteric microbiome. Compared to their parent compounds, these metabolites exert much better anticancer activities with multiple molecular mechanisms. Interestingly, both Rg3 and CK also showed significant anti-angiogenic effects on pulmonary, gastric, and ovarian cancers. Therefore, in addition to the chemopreventive effects of ginseng compounds, as angiogenic inhibitors, ginsenoside metabolites could be used in combination with other cancer chemotherapeutic agents in cancer management.

## References

[1] Siegel R.L., Miller K.D., Jemal A. (2015). Cancer statistics, 2015. CA Cancer J. Clin..

[2] De Santis C.E., Lin C.C., Mariotto A.B., Siegel R.L., Stein K.D., Kramer J.L., Alteri R., Robbins A.S., Jemal A. (2014). Cancer treatment and survivorship statistics, 2014. CA Cancer J. Clin..

[3] Hesketh P.J. (2008). Chemotherapy-induced nausea and vomiting. N. Engl. J. Med..

[4] Meador C.B., Micheel C.M., Levy M.A., Lovly C.M., Horn L., Warner J.L., Johnson D.B., Zhao Z., Anderson I.A., Sosman J.A. (2014). Beyond histology: Translating tumor genotypes into clinically effective targeted therapies. Clin. Cancer Res..

[5] Biselli-Chicote P.M., Oliveira A.R., Pavarino E.C., Goloni-Bertollo E.M. (2012). VEGF gene alternative splicing: Pro- and anti-angiogenic isoforms in cancer. J. Cancer Res. Clin. Oncol..

[6] Wilson W.R., Hay M.P. (2011). Targeting hypoxia in cancer therapy. Nat. Rev. Cancer.

[7] Wang Y.Q., Miao Z.H. (2013). Marine-derived angiogenesis inhibitors for cancer therapy. Mar. Drugs.

[8] Davis E.L., Oh B., Butow P.N., Mullan B.A., Clarke S. (2012). Cancer patient disclosure and patient-doctor communication of complementary and alternative medicine use: A systematic review. Oncologist.

[9] Wang C.Z., Calway T., Yuan C.S. (2012). Herbal medicines as adjuvants for cancer therapeutics. Am. J. Chin. Med..

[10] Wang C.Z., He H., Wang X., Yuan C.S. (2012). Trends in scientific publications of Chinese medicine. Am. J. Chin. Med..

[11] Bell R.M. (2010). A review of complementary and alternative medicine practices among cancer survivors. Clin. J. Oncol. Nurs..

[12] Lin J.G., Chen Y.H. (2012). The role of acupuncture in cancer supportive care. Am. J. Chin. Med..

[13] Hait W.N., Hambley T.W. (2009). Targeted cancer therapeutics. Cancer Res..

[14] Xu Z., Chen X., Zhong Z., Chen L., Wang Y. (2011). Ganoderma lucidum polysaccharides: Immunomodulation and potential anti-tumor activities. Am. J. Chin. Med..

[15] Lee J., Chae J., Lee S., Kim K., Eo W., Kim S., Choi W., Cheon S.H. (2013). The efficacy and safety of standardized allergen-removed Rhus verniciflua extract as maintenance therapy after first-line chemotherapy in patients with advanced non-small cell lung cancer. Am. J. Chin. Med..

[16] Cragg G.M., Grothaus P.G., Newman D.J. (2009). Impact of natural products on developing new anti-cancer agents. Chem. Rev..

[17] Sousa T., Paterson R., Moore V., Carlsson A., Abrahamsson B., Basit A.W. (2008). The gastrointestinal microbiota as a site for the biotransformation of drugs. Int. J. Pharm..

[18] Haiser H.J., Turnbaugh P.J. (2012). Is it time for a metagenomic basis of therapeutics?. Science.

[19] Shergis J.L., Zhang A.L., Zhou W., Xue C.C. (2013). Quality and risk of bias in Panax ginseng randomized controlled trials: A review. Am. J. Chin. Med..

[20] Gu C., Qiao J., Zhu M., Du J., Shang W., Yin W., Wang W., Han M., Lu W. (2013). Preliminary evaluation of the interactions of Panax ginseng and Salvia miltiorrhiza Bunge with 5-fluorouracil on pharmacokinetics in rats and pharmacodynamics in human cells. Am. J. Chin. Med..

[21] Wang C.Z., Ni M., Sun S., Li X.L., He H., Mehendale S.R., Yuan C.S. (2009). Detection of adulteration of notoginseng root extract with other panax species by quantitative HPLC coupled with PCA. J. Agric. Food Chem..

[22] Xu Y., Lin L., Tang L., Zheng M., Ma Y., Huang L., Meng W., Wang W. (2014). Notoginsenoside R1 attenuates hypoxia and hypercapnia-induced vasoconstriction in isolated rat pulmonary arterial rings by reducing the expression of ERK. Am. J. Chin. Med..

[23] Wang C.Z., Kim K.E., Du G.J., Qi L.W., Wen X.D., Li P., Bauer B.A., Bissonnette M.B., Musch M.W., Chang E.B. (2011). Ultra-performance liquid chromatography and time-of-flight mass spectrometry analysis of ginsenoside metabolites in human plasma. Am. J. Chin. Med..

[24] Park E.Y., Kim M.H., Kim E.H., Lee E.K., Park I.S., Yang D.C., Jun H.S. (2014). Efficacy comparison of Korean ginseng and American ginseng on body temperature and metabolic parameters. Am. J. Chin. Med..

[25] Yu C., Wang C.Z., Zhou C.J., Wang B., Han L., Zhang C.F., Wu X.H., Yuan C.S. (2014). Adulteration and cultivation region identification of American ginseng using HPLC coupled with multivariate analysis. J. Pharm. Biomed. Anal..

[26] Fuzzati N. (2004). Analysis methods of ginsenosides. J. Chromatogr. B.

[27] Chao Z., Shoyama Y., Tanaka H. (2006). Pharmacokinetic study of ginsenosides Rb1 and Rg1 in rat by ELISA using anti-ginsenosides Rb1 and Rg1 monoclonal antibodies. Am. J. Chin. Med..

[28] Wang C.Z., McEntee E., Wicks S., Wu J.A., Yuan C.S. (2006). Phytochemical and analytical studies of Panax notoginseng (Burk.) F.H. Chen. J. Nat. Med..

[29] Qi L.W., Wang C.Z., Yuan C.S. (2011). Isolation and analysis of ginseng: Advances and challenges. Nat. Prod. Rep..

[30] Sun S., Qi L.W., Du G.J., Mehendale S.R., Wang C.Z., Yuan C.S. (2011). Red notoginseng: Higher ginsenoside content and stronger anticancer potential than Asian and American ginseng. Food Chem..

[31] Wang C.Z., Aung H.H., Ni M., Wu J.A., Tong R., Wicks S., He T.C., Yuan C.S. (2007). Red American ginseng: Ginsenoside constituents and antiproliferative activities of heat-processed Panax quinquefolius roots. Planta Med..

[32] Zhang Y.L., Zhang R., Xu H.L., Yu X.F., Qu S.C., Sui D.Y. (2013). 20(*S*)-protopanaxadiol triggers mitochondrial-mediated apoptosis in human lung adenocarcinoma A549 cells via inhibiting the PI3K/Akt signaling pathway. Am. J. Chin. Med..

[33] Yuan C.S., Dey L. (2001). Multiple effects of American ginseng in clinical medicine. Am. J. Chin. Med..

[34] Valli G., Giardina E.G. (2002). Benefits, adverse effects and drug interactions of herbal therapies with cardiovascular effects. J. Am. Coll. Cardiol..

[35] Attele A.S., Zhou Y.P., Xie J.T., Wu J.A., Zhang L., Dey L., Pugh W., Rue P.A., Polonsky K.S., Yuan C.S. (2002). Antidiabetic effects of Panax ginseng berry extract and the identification of an effective component. Diabetes.

[36] Xie J.T., Aung H.H., Wu J.A., Attele A.S., Yuan C.S. (2002). Effects of American ginseng berry extract on blood glucose levels in *ob*/*ob* mice. Am. J. Chin. Med..

[37] Xie J.T., Zhou Y.P., Dey L., Attele A.S., Wu J.A., Polonsky K.S., Yuan C.S. (2002). *Panax ginseng* berry extract reduces blood glucose and body weight in *db*/*db* mice. Phytomedicine.

[38] Zhan Y., Xu X.H., Jiang Y.P. (1994). Protective effects of ginsenoside on myocardiac ischemic and reperfusion injuries. Zhonghua Yi Xue Za Zhi.

[39] Shao Z.H., Xie J.T., Vanden Hoek T.L., Mehendale S., Aung H., Li C.Q., Qin Y., Schumacker P.T., Becker L.B., Yuan C.S. (2004). Antioxidant effects of American ginseng berry extract in cardiomyocytes exposed to acute oxidant stress. Biochim. Biophys. Acta.

[40] Mehendale S.R., Aung H.H., Yin J.J., Lin E., Fishbein A., Wang C.Z., Xie J.T., Yuan C.S. (2004). Effects of antioxidant herbs on chemotherapy-induced nausea and vomiting in a rat-pica model. Am. J. Chin. Med..

[41] Mehendale S., Aung H., Wang A., Yin J.J., Wang C.Z., Xie J.T., Yuan C.S. (2005). American ginseng berry extract and ginsenoside Re attenuate cisplatin-induced kaolin intake in rats. Cancer Chemother. Pharmacol..

[42] Compare D., Nardone G. (2011). Contribution of gut microbiota to colonic and extracolonic cancer development. Digit. Dis..

[43] Sekirov I., Russell S.L., Antunes L.C., Finlay B.B. (2010). Gut microbiota in health and disease. Physiol. Rev..

[44] Chi H., Ji G.E. (2005). Transformation of ginsenosides Rb1 and Re from Panax ginseng by food microorganisms. Biotechnol. Lett..

[45] Hasegawa H., Sung J.H., Benno Y. (1997). Role of human intestinal Prevotella oris in hydrolyzing ginseng saponins. Planta Med..

[46] Wan J.Y., Liu P., Wang H.Y., Qi L.W., Wang C.Z., Li P., Yuan C.S. (2013). Biotransformation and metabolic profile of American ginseng saponins with human intestinal microflora by liquid chromatography quadrupole time-of-flight mass spectrometry. J. Chromatogr. A.

[47] Wang C.Z., Du G.J., Zhang Z., Wen X.D., Calway T., Zhen Z., Musch M.W., Bissonnette M., Chang E.B., Yuan C.S. (2012). Ginsenoside compound K, not Rb1, possesses potential chemopreventive activities in human colorectal cancer. Int. J. Oncol..

[48] Bae E.A., Choo M.K., Park E.K., Park S.Y., Shin H.Y., Kim D.H. (2002). Metabolism of ginsenoside R(c) by human intestinal bacteria and its related antiallergic activity. Biol. Pharm. Bull..

[49] Wang C.Z., Aung H.H., Zhang B., Sun S., Li X.L., He H., Xie J.T., He T.C., Du W., Yuan C.S. (2008). Chemopreventive effects of heat-processed Panax quinquefolius root on human breast cancer cells. Anticancer Res..

[50] Lee J., Lee E., Kim D., Yoo J., Koh B. (2009). Studies on absorption, distribution and metabolism of ginseng in humans after oral administration. J. Ethnopharmacol..

[51] Tawab M.A., Bahr U., Karas M., Wurglics M., Schubert-Zsilavecz M. (2003). Degradation of ginsenosides in humans after oral administration. Drug Metab. Dispos..

[52] Li N., Wang D., Ge G., Wang X., Liu Y., Yang L. (2014). Ginsenoside metabolites inhibit p-glycoprotein *in vitro* and *in situ* using three absorption models. Planta Med..

[53] Gao J.L., Lv G.Y., He B.C., Zhang B.Q., Zhang H., Wang N., Wang C.Z., Du W., Yuan C.S., He T.C. (2013). Ginseng saponin metabolite 20(*S*)-protopanaxadiol inhibits tumor growth by targeting multiple cancer signaling pathways. Oncol. Rep..

[54] Wang C.Z., Li B., Wen X.D., Zhang Z., Yu C., Calway T.D., He T.C., Du W., Yuan C.S. (2013). Paraptosis and NF-kappaB activation are associated with protopanaxadiol-induced cancer chemoprevention. BMC Complement. Altern. Med..

[55] Lu P., Su W., Miao Z.H., Niu H.R., Liu J., Hua Q.L. (2008). Effect and mechanism of ginsenoside Rg3 on postoperative life span of patients with non-small cell lung cancer. Chin. J. Integr. Med..

[56] Keum Y.S., Han S.S., Chun K.S., Park K.K., Park J.H., Lee S.K., Surh Y.J. (2003). Inhibitory effects of the ginsenoside Rg3 on phorbol ester-induced cyclooxygenase-2 expression, NF-kappaB activation and tumor promotion. Mutat. Res..

[57] Kim S.M., Lee S.Y., Cho J.S., Son S.M., Choi S.S., Yun Y.P., Yoo H.S., Yoon Do Y., Oh K.W., Han S.B. (2010). Combination of ginsenoside Rg3 with docetaxel enhances the susceptibility of prostate cancer cells via inhibition of NF-kappaB. Eur. J. Pharmacol..

[58] He B.C., Gao J.L., Luo X., Luo J., Shen J., Wang L., Zhou Q., Wang Y.T., Luu H.H., Haydon R.C. (2011). Ginsenoside Rg3 inhibits colorectal tumor growth through the down-regulation of Wnt/ss-catenin signaling. Int. J. Oncol..

[59] Cho S.H., Chung K.S., Choi J.H., Kim D.H., Lee K.T. (2009). Compound K, a metabolite of ginseng saponin, induces apoptosis via caspase-8-dependent pathway in HL-60 human leukemia cells. BMC Cancer.

[60] Kim do Y., Park M.W., Yuan H.D., Lee H.J., Kim S.H., Chung S.H. (2009). Compound K induces apoptosis via CAMK-IV/AMPK pathways in HT-29 colon cancer cells. J. Agric. Food Chem..

[61] Kim do Y., Yuan H.D., Chung I.K., Chung S.H. (2009). Compound K, intestinal metabolite of ginsenoside, attenuates hepatic lipid accumulation via AMPK activation in human hepatoma cells. J. Agric. Food Chem..

[62] Choo M.K., Sakurai H., Kim D.H., Saiki I. (2008). A ginseng saponin metabolite suppresses tumor necrosis factor-alpha-promoted metastasis by suppressing nuclear factor-kappaB signaling in murine colon cancer cells. Oncol. Rep..

[63] Park S., Lee H.J., Jeong S.J., Song H.S., Kim M., Lee E.O., Kim D.H., Ahn K.S., Kim S.H. (2011). Inhibition of JAK1/STAT3 signaling mediates compound K-induced apoptosis in human multiple myeloma U266 cells. Food Chem. Toxicol..

[64] Kim A.D., Kang K.A., Kim H.S., Kim D.H., Choi Y.H., Lee S.J., Hyun J.W. (2013). A ginseng metabolite, compound K, induces autophagy and apoptosis via generation of reactive oxygen species and activation of JNK in human colon cancer cells. Cell Death Dis..

[65] Zhang Z., Du G.J., Wang C.Z., Wen X.D., Calway T., Li Z., He T.C., Du W., Bissonnette M., Musch M.W. (2013). Compound K, a Ginsenoside Metabolite, Inhibits Colon Cancer Growth via Multiple Pathways Including p53–p21 Interactions. Int. J. Mol. Sci..

[66] Mochizuki M., Yoo Y.C., Matsuzawa K., Sato K., Saiki I., Tono-oka S., Samukawa K., Azuma I. (1995). Inhibitory effect of tumor metastasis in mice by saponins, ginsenoside-Rb2, 20(*R*)- and 20(*S*)-ginsenoside-Rg3, of red ginseng. Biol. Pharm. Bull..

[67] Kerbel R.S. (2008). Tumor angiogenesis. N. Engl. J. Med..

[68] Yue P.Y., Wong D.Y., Wu P.K., Leung P.Y., Mak N.K., Yeung H.W., Liu L., Cai Z., Jiang Z.H., Fan T.P. (2006). The angiosuppressive effects of 20(*R*)-ginsenoside Rg3. Biol. Pharmacol..

[69] Chen Q.J., Zhang M.Z., Wang L.X. (2010). Gensenoside Rg3 inhibits hypoxia-induced VEGF expression in human cancer cells. Cell. Physiol. Biochem..

[70] Yu Y., Zhang C., Liu L., Li X. (2013). Hepatic arterial administration of ginsenoside Rg3 and transcatheter arterial embolization for the treatment of VX2 liver carcinomas. Exp. Ther. Med..

[71] Yu H., Teng L., Meng Q., Li Y., Sun X., Lu J., Lee R.J., Teng L. (2013). Development of liposomal Ginsenoside Rg3: Formulation optimization and evaluation of its anticancer effects. Int. J. Pharm..

[72] Liu T.G., Huang Y., Cui D.D., Huang X.B., Mao S.H., Ji L.L., Song H.B., Yi C. (2009). Inhibitory effect of ginsenoside Rg3 combined with gemcitabine on angiogenesis and growth of lung cancer in mice. BMC Cancer.

[73] Zhang Q., Kang X., Zhao W. (2006). Antiangiogenic effect of low-dose cyclophosphamide combined with ginsenoside Rg3 on Lewis lung carcinoma. Biochem. Biophys. Res. Commun..

[74] Zhang Q., Kang X., Yang B., Wang J., Yang F. (2008). Antiangiogenic effect of capecitabine combined with ginsenoside Rg3 on breast cancer in mice. Cancer Biother. Radiopharm..

[75] Lin S.S., Lai K.C., Hsu S.C., Yang J.S., Kuo C.L., Lin J.P., Ma Y.S., Wu C.C., Chung J.G. (2009). Curcumin inhibits the migration and invasion of human A549 lung cancer cells through the inhibition of matrix metalloproteinase-2 and -9 and Vascular Endothelial Growth Factor (VEGF). Cancer Lett..

[76] Sawaya R., Go Y., Kyritisis A.P., Uhm J., Venkaiah B., Mohanam S., Gokaslan Z.L., Rao J.S. (1998). Elevated levels of Mr 92,000 type IV collagenase during tumor growth *in vivo*. Biochem. Biophys. Res. Commun..

[77] Jung S.H., Woo M.S., Kim S.Y., Kim W.K., Hyun J.W., Kim E.J., Kim D.H., Kim H.S. (2006). Ginseng saponin metabolite suppresses phorbol ester-induced matrix metalloproteinase-9 expression through inhibition of activator protein-1 and mitogen-activated protein kinase signaling pathways in human astroglioma cells. Int. J. Cancer.

[78] Jeong A., Lee H.J., Jeong S.J., Lee E.O., Bae H., Kim S.H. (2010). Compound K inhibits basic fibroblast growth factor-induced angiogenesis via regulation of p38 mitogen activated protein kinase and AKT in human umbilical vein endothelial cells. Biol. Pharm. Bull..

[79] Shin K.O., Seo C.H., Cho H.H., Oh S., Hong S.P., Yoo H.S., Hong J.T., Oh K.W., Lee Y.M. (2014). Ginsenoside compound K inhibits angiogenesis via regulation of sphingosine kinase-1 in human umbilical vein endothelial cells. Arch. Pharm. Res..

